# Impact of a Long Lockdown on Mental Health and the Role of Media Use: Web-Based Survey Study

**DOI:** 10.2196/36050

**Published:** 2022-06-28

**Authors:** Dominika Grygarová, Petr Adámek, Veronika Juríčková, Jiří Horáček, Eduard Bakštein, Iveta Fajnerová, Ladislav Kesner

**Affiliations:** 1 Center for Advanced Studies of Brain and Consciousness National Institute of Mental Health Klecany Czech Republic; 2 Third Faculty of Medicine Charles University Prague Czech Republic; 3 First Faculty of Medicine Charles University Prague Czech Republic; 4 Faculty of Electrical Engineering Czech Technical University in Prague Prague Czech Republic; 5 Early Stages of Severe Mental Illness Research Center National Institute of Mental Health Klecany Czech Republic; 6 Virtual Reality in Mental Health and Neuroscience Research Center National Institute of Mental Health Klecany Czech Republic; 7 Faculty of Arts Masaryk University Brno Czech Republic

**Keywords:** mental health, COVID-19, lockdown, media use, anxiety, depression, nationally representative data, survey, longitudinal study, pandemic, social isolation, social media, psychological trauma, mental stress, media news

## Abstract

**Background:**

Due to the COVID-19 pandemic, the Czech population experienced a second lockdown lasting for about half a year, restricting free movement and imposing social isolation. However, it is not known whether the impact of this long lockdown resulted in habituation to the adverse situation or in the traumatization of the Czech population, and whether the media and specific media use contributed to these effects.

**Objective:**

The aim of this study was to elucidate the effect of the long lockdown on the mental health of the Czech population, and the role of exposure to COVID-19 news reports and specific forms of media news use in mental health.

**Methods:**

We conducted two consecutive surveys in the early (November 2020) and late (March/April 2021) phases of the nationwide lockdown on the same nationally representative group of Czech adults (N=1777) participating in a longitudinal panel study.

**Results:**

Our findings showed that the self-reported symptoms of anxiety and depression increased in the second observation period, confirming the negative effect of the pandemic lockdown as it unfolded, suggesting that restrictive measures and continuous exposure to a collective stressor did not result in the strengthening of resilience but rather in ongoing traumatization. The results also suggest a negative role of the media’s coverage of the COVID-19 pandemic in mental health during the early, and particularly late, phases of the lockdown. Furthermore, we found several risk and protective factors of specific media news use. The media practice in news consumption connected to social media use was the strongest predictor of exacerbated mental health symptoms, particularly in the late phase of the lockdown. Moreover, news media use characterized by internalization of information learned from the news, as well as negative attitudes toward media news, were associated with higher levels of anxiety and depression. Conversely, the use of infotainment, together with an in-depth and contextual style of reading news articles, were related to improvement of mental health.

**Conclusions:**

Our study showed that the long lockdown resulted in traumatization rather than habituation, and in more pronounced effects (both negative and positive) of media use in mental health.

## Introduction

### The Role of Media Use During the COVID-19 Pandemic in Mental Health

As the global COVID-19 pandemic has gradually evolved since its inception early in 2020, it has been increasingly apparent that it constitutes not only an unprecedented epidemiological and medical emergency but also a major psychological, social, and political problem. Since the outbreak of the epidemic, numerous studies have examined its impact on measures of the mental health and well-being of the populations of many countries [1], including Czechia [2].

Early on, it was also recognized that perception of the pandemic and its impact on mental well-being were to a substantial extent determined by the ways the media covered the course of the epidemic [3,4]. To date, research on media use in relation to mental health has focused primarily on the exposure to traumatic news and the use of social media. Previous research established that exposure to media reporting of traumatic events such as terrorist attacks or wars exacerbates subjective measures of mental health, particularly posttraumatic stress disorder, anxiety, and depressive symptoms [5-8]. Analogous results were obtained in research examining the media effects in nonclinical populations in the context of the COVID-19 pandemic. Several studies analyzed participants’ subjective measures of mental health in relation to certain aspects of their news consumption, primarily exposure to news reports or specifically to COVID-19 news reports [9-13]. Another large group of studies examined the use of social media [14-16]. Across these studies, higher frequencies of both news consumption and social media use were consistently found as factors connected to (self-reported) poorer mental health.

The inherent limitation of this body of research may be related to the fact that it examined the effects of only one or two chosen indicators of media use. Therefore, some important information on specific media effects on mental health, as well as internal relationships between particular dimensions of media behavior, may be lacking. Hence, when potentially adverse effects of media use on mental health are reduced to only one dimension of media behavior (such as the exposure to news reports), erroneous generalizations may ensue (eg, media news consumption as such is solely harmful to mental health).

In media theory, analogous limitations of the dominant quantitative approach to media research have been identified by the media repertoires approach [17]. This theory criticizes traditional media research methodology for being descriptive of only one single studied media type, thereby not being able to adequately analyze people’s actual everyday practices. In contrast, the media repertoires approach aims at studying individual patterns of media use, including a composition of different media types or technologies, different content, the way they are consumed, and how these repertoires are interrelated [18]. In the context of the COVID-19 pandemic, Pahayahay et al [19] used this approach to assess both the harms and benefits of screen media use as a coping mechanism for self-isolation. However, it is still unknown whether some forms of media news use have the potential to positively impact mental well-being or offer a possible remedy for “safer” media news use. Such an understanding is crucial in terms of developing recommendations for media coverage of a crisis and in terms of media hygiene recommendations for media users.

To address this gap, we decided to relate subjective measures of mental health to more realistic and comprehensive media news use patterns extracted from our data. Therefore, we performed a representative survey covering two periods of the pandemic lockdown, taking into account a number of indicators of media behavior, namely the total time of media news consumption, type of news content, frequency of social media use as a source of information, type of media, category of media (eg, public, commercial, antisystem), level of detail to which the news is read (eg, only headlines, full news articles), reading comments in discussions below news articles, subjective attitudes to the media, perceived stress from the media, and internalization of media news. Inspired by the media repertoires approach, we were further interested in whether there is a latent structure behind these forms of media use that would reveal interrelated practices of media news use, and whether some of them are risk or protective factors for mental health.

### COVID-19 and its Media Representations as a Dynamic Stressor

At least as of late spring 2020, it become apparent that the COVID-19 pandemic represented a dynamically evolving stressor, and that the context of potential media impacts on mental health were changing over time in response to both global and locally specific patterns of pandemic development and the societal response to it. The next step in understanding the role of the media in the COVID-19 crisis was to determine whether media coverage of the COVID-19 pandemic played the same role in mental health of the population during the different phases of the pandemic to determine whether media representations of the pandemic represent a dynamic stressor. In addition, comparing associations between other specific media practices and mental health in two different phases would reveal the dynamic aspect of their negative/positive role in mental health. To the best of our knowledge, no previous research has examined the link between media news use and mental health in a longitudinal design on a representative sample. We set out to examine associations between mental health (depression, anxiety) and media use in a population-level representative survey using a longitudinal design, collecting responses in two subsequent waves from the same group of participants in Czechia.

**Figure 1:**
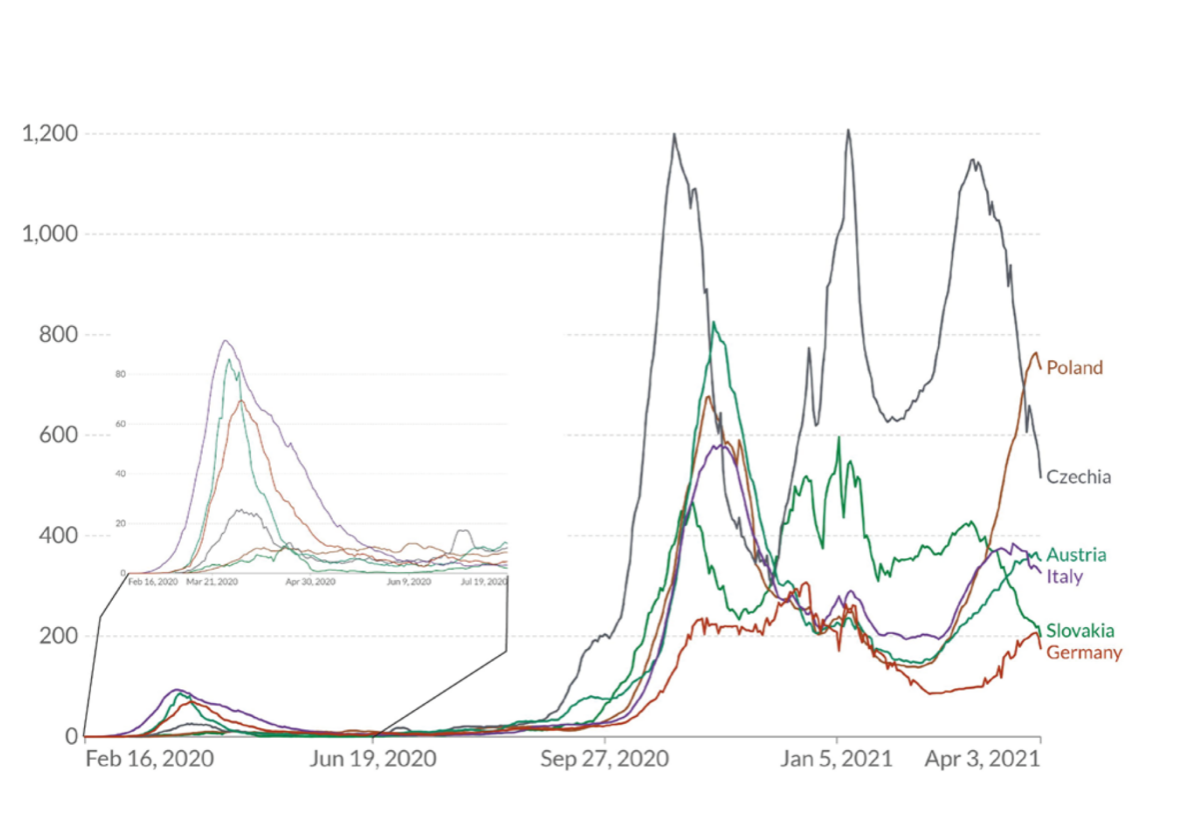
Visualization of daily new confirmed COVID-19 cases per million people in Czechia. In comparison to surrounding countries (Poland, Germany, Austria, Slovakia) and Italy, Czechia was the worst affected European country at the peak of the first wave of the COVID-19 pandemic. Visualization and data retrieved from Johns Hopkins University [20].

The first wave of our survey took place at the beginning of November 2020, the second week after a second nationwide lockdown was announced, imposing social isolation; restriction of free movement; and the closure of schools, restaurants, and most shops. The lockdown was declared when Czechia became the worst affected country in the world, with the highest per capita incidence and mortality related to COVID-19 (Figure 1) [20]. The epidemiological situation deteriorated after the summer of 2020, when almost all protective measures maintained after the first nationwide lockdown in the spring of 2020 were abandoned to an extent without parallel elsewhere. Therefore, the first wave of our survey took place at the highest point of the crisis, in the week when the highest daily incidence had been reached with 15,726 newly reported cases, and the daily mortality count reached its peak with 261 reported fatalities [20]. The second wave of the survey took place at the end of March and beginning of April 2021, about 2 weeks after the peak of the third wave and roughly 5 months since the beginning of the lockdown in November 2020, with only a brief release of restrictive measures during a slight decline in the epidemic in December 2020. During the week of our second survey, the highest daily incidence of the second wave had been reached, with 8664 newly reported cases, and the daily mortality count reached its peak with the 218 reported fatalities (Figure 1) [20]. Together, our survey covered two periods of maximal pandemic outbreak, characterized by comparable objective measures of the pandemic situation (extreme daily incidence and mortality); although the epidemiological situation was slightly more favorable in the second wave of the survey, this was not the case for the social climate and public mood.

Therefore, compared to previous longitudinal studies that examined the change in mental health between the period before and during the COVID-19 pandemic [21], or the effect of a nationwide lockdown lasting 1 or 2 months [22], the design of our study allowed us to observe the effect of a continuous 5-month-long lockdown spanning a continuing public health and social crisis.

### Aims of the Research

The first aim of our study was to compare the levels of anxiety and depression of the Czech population between the early and late phases of the COVID-19 lockdown. We hypothesized that the continuing influx of traumatic news, along with the negative impact of long-term uncertainty, possible increasing frustration, and resistance toward government measures will result in an increase in self-reported symptoms of anxiety and depression. Second, we were interested in whether consumption of media coverage of COVID-19 played a role in mental health and whether/how it changed between the two observation periods. The third aim was to specify more complex media use practices and their role in mental health in both observation periods.

## Methods

### Procedure

Data were collected during two phases of the nationwide pandemic lockdown in Czechia (first survey: November 2-8, 2020; second survey: March 29 to April 6, 2021). Our two surveys were completed by a representative cohort within the Czech National Panel [23] using a standardized computer-assisted web interviewing method. The mean completion time of the survey was 8 minutes 25 seconds. The survey comprised demographic data (gender, age, level of education, region of residence, and household income), standardized mental health measures, and our comprehensive Media Use Questionnaire (MUQ). Only self-reported measures were used.

### Ethics Approval

The study was approved by the Ethics Committee of the National Institute of Mental Health, Czechia (project number 115/19), and all participants provided informed consent. The participation was voluntary with a financial reward within the Czech National Panel.

### Participants

In the first and second waves of the survey, we obtained answers from 2214 (return rate 71%) and 2061 (return rate 67%) respondents, respectively, with 86% of the respondents in the second wave also participating in the first wave. Thus, the final study sample consisted of 1777 individuals (n=890 women, 50.08%) aged between 18 and 91 years (mean 53.06, SD 15.89 years). The distribution in educational level of the participants was 4.9% elementary school education, 26.3% certificate of apprenticeship, 37.5% high school education, and 31.3% university degree. The inclusion criteria were age above 18 years and knowledge of the Czech language.

### Measures

#### Mental Health

The 8-item Patient Health Questionnaire Depression Scale (PHQ-8) [24] was used to measure depression severity within the past 2 weeks. The PHQ-8 score was standardly divided into five levels: no (0-4 points), mild (5-9), moderate (10-14), moderate to severe (15-19), and severe (20-24) depressive symptoms. The Generalized Anxiety Disorder questionnaire (GAD-7) [25] was used to quantify the degree of anxiety. The GAD-7 score was standardly divided into five levels: minimal (0-4), mild (5-9), moderate (10-14), and severe (15-21) anxiety symptoms.

#### Media Use

We developed a comprehensive Media Use Questionnaire (MUQ) mapping eight major indicators of media use within the past 2 weeks based on 33 items, as summarized in Textbox 1.

**Main indicators of the Media Use Questionnaire (MUQ)**

- *Exposure to media news*

Respondents were asked to estimate the time of daily consumption of news in the media in hours and minutes (television, radio, press, and internet, including social networks).

- *Media categories*

Thirty-six Czech media outlets with predominant news content were sorted into five categories: “public,” “mainstream,” “commercial,” “antisystem,” “information from official public sources” (such as the Ministry of Health), and “opinion online newspapers” claiming a specific political orientation. Participants indicated the frequency of use of each media category on a 5-point Likert scale ranging from “never” to “several times a day–more than two times.” These media categories were further divided into two types: *audiovisual/audio* (passive use: watching/listening) and *print/internet* (active use: reading). The frequency of their use was calculated as a mean of the summary of frequencies of the usage of each corresponding media category.

- *News content*

News was divided into several categories based on the content: politics, economics, COVID-19 pandemic, entertainment/show business, culture, sport, science and technologies, crime, transport, weather, environment, and health. Participants rated how much they were interested in each topic on a 4-point scale (from “I was not interested at all” to “I was very interested”).

- *Reading comments*

Participants were asked to rate how frequently they read online comments in discussions under web news articles on a 5-point scale from “never” to “very often.”

- *Social media as an information source*

Participants evaluated how often they use social media as a means of obtaining news information on a 5-point scale from “never” to “very often.”

- *Reading habits*

Participants rated how thoroughly they read the news reports on a 6-point scale (1: “I do not read news,” 2: “I usually read only headlines,” 3: “I usually read only headlines and first few sentences,” 4: “I usually read half of the article,” 5: “I usually read the whole article,” 6: “I usually read the whole article and I search for further information on the discussed issue”).

- *Perceived impact of media news*

(1) *Perceived stress* from the media news was evaluated using a questionnaire taken from Liu and Liu [3].

(2) *Internalization of media news* was accessed with four statements assessing whether the information one learns in the news was internalized in one’s thoughts, interactions with others, or whether it influences one’s attitudes or behavior.

Responses on both scales were rated on a 4-point scale from “strongly disagree” to “strongly agree.”

- *Attitudes to the media*

Participants were asked to rate various statements about media expressing *mistrust* (“I do not trust media”), *frustration* (“Media news usually frustrates or upsets me”), *annoyance* (“It seems that the news keeps saying the same thing over and over”), *stress avoidance* (“I do not want to be unnecessarily stressed by the news”), *lack of interest* (“I am not usually interested in the news”), *lack of concentration* (“I usually cannot concentrate on the news”), *lack of time* (“I don’t have time to watch/read/listen to the news”), *positive appreciation* (“The information I learn in the media news helps me find my way around today’s world”). The responses were rated on a 6-point scale from “strongly disagree” to “strongly agree.”

### Statistical Analysis

All data were analyzed using R software [26]. The significance level was set to *P*<.05. Poststratification weighting was performed using a quadratic programming algorithm given the current population distributions of the following characteristics: gender, age, education, region and residence size, job status and description, interaction between age and education, and interaction between age and gender.

Descriptive statistics were used for demographics. As the Shapiro-Wilk test did not confirm a normal distribution, we used the nonparametric Wilcoxon signed-rank test with continuity correction to evaluate the differences in mental health and media use between the two waves of data collection. Exploratory factor analysis (EFA) was performed to reduce the MUQ data and to show its latent structure based on interdependencies between the items. The Bartlett test of sphericity and the Kaiser-Meyer-Olkin measure of sampling adequacy indicated the suitability of our data for structure detection. Principal component analysis identified seven factors with an eigenvalue greater than 1. Direct Oblimin rotation not requiring orthogonality of the factors was used. Factor scores were computed for each observer. Confirmatory factor analysis was performed for media use data accessed from the MUQ from the second wave to confirm the first-wave EFA factors and to calculate the factor score.

Multiple linear regression models were used to reveal the relationships between media use factors and mental health. For the linear models, we used normalization of nonparametric right-skewed data by square-root transformation. Separate models for anxiety and depression (dependent variables) in both observation periods of the pandemic lockdown were calculated and controlled for demographic characteristics (age, gender). The seven media factors extracted from EFA were further used in multiple linear regression models as predictors. Media variables that did not saturate any of the factors (COVID-19 news exposure, media news exposure, reading habits) were used in the linear models as separate explanatory variables.

## Results

### Mental Health

The majority of the sample reported no/minimal anxiety and depression during both waves of the survey (Table 1). The results of the Wilcoxon signed-rank test showed a significant increase in anxiety (V=208,263, *P*=.005) and depression (V=256,681, *P*<.001) levels between the two waves. Higher levels of anxiety and depression were associated with female gender and younger age in both waves. The anxiety and depression scores were highly correlated, showing a Pearson correlation coefficient of *r*=0.889 and *r*=0.895 in the first and second wave, respectively.

**Table 1:** Scores of the Generalized Anxiety Disorder questionnaire (GAD-7) and Patient Health Questionnaire Depression Scale (PHQ-8) in the first and second waves of the survey (N=1771).

Scale		First wave, n (%)	Second wave, n (%)
**GAD-7**			
	Minimal symptoms	1420 (79.9)	1377 (77.75)
	Mild	248 (14)	280 (15.8)
	Moderate	77 (4.3)	66 (3.7)
	Severe	32 (1.8)	54 (3.0)
**PHQ-8**			
	No symptoms	1330 (74.8)	1308 (73.6)
	Mild	300 (16.9)	304 (17.1)
	Moderate	94 (5.3)	90 (5.1)
	Moderate to severe	37 (2.1)	51 (2.9)
	Severe	16 (1.4)	24 (1.4)

### Media Use

The frequencies of the answers to the individual MUQ items are presented in Multimedia Appendix 1. We found significant differences between the two waves in several media variables (social media as news source; reading comments; use of commercial, mainstream, audio/audiovisual, print/internet media; reading habits; perceived stress from media news; internalization of media news; positive appreciation of media news; negative attitude to the news: lack of interest; use of the following news sections: politics, economics, COVID-19–related news, entertainment/show business, culture, science and technologies, crime, transport, weather, environment, and health). The rates in these dimensions of media behavior decreased except for the use of print/internet news and lack of interest in the news, which increased. In overall exposure to media news, we found no significant difference between the two waves.

### Media Use Factors

EFA extracted seven factors with an eigenvalue greater than 1 (Table 2). The overall value of the Kaiser-Meyer-Olkin measure of sampling adequacy (0.83) indicated a good level of suitability for analysis. The Bartlett sphericity test (*χ^2^*_435_=16,995.02, *P*=.001) also showed that the items in the MUQ are related and suitable for factor analysis. Multimedia Appendix 2 shows the factor loadings of the seven media factors.

**Table 2:** Media factors, factor items, and factor descriptions.

Media factor (MF)	Factor items	Factor description
MF1: Conscientious public news use	Lack of interest in news (negative factor loading), lack of time to follow news (negative factor loading), politics, economics, public media (audiovisual broadcast media)	Media practice characterized by interest in media news and determination to find time and follow information of great social significance (“hard news” other than pandemic-related) via public media (the media outlet commonly viewed as the most reliable)
MF2: News aversion	Frustration with news, annoyance with news, stress avoidance, mistrust in media, lack of concentration to news	The use of media that is characterized by various negative attitudes to the media: mistrust in the media; frustration, annoyance, and avoidance behavior toward the media news
MF3: News reading	Mainstream media, foreign media, antisystem media, official public sources, opinion online newspapers	Media practice characterized by the use of news from various media categories other than public and commercial (which are mostly audio/audiovisual). Media news reports in MF3 have a variety of journalistic styles but are almost exclusively of the print/internet type (requiring reading)
MF4: Social media practice in news consumption	Reading comments, social media as a news source, perceived stress from the media	Media practice of news consumption linked to social media use characterized by using social media as an information source, reading comments, and subjectively perceived stress from the media
MF5: Infotainment	Commercial media, entertainment, crime, sports	Media practice characterized by the use of commercial media news and the use of entertainment topics that are typical for commercial media (show business, sports, and crime)
MF6: Internalized use	Internalization of media news, positive appreciation of news	Media practice that attaches great importance to news information, which has a great impact on personal inner/social life and behavior. This practice also groups a positive appreciation of media news (the impact of information on one’s own life must necessarily be associated with some level of trust in the source of the information)
MF7: Use of practical news	Transport, weather, environment, health, culture, science and technologies	Media practice characterized by the use of news sections providing practical information, which helps to navigate daily life

### Associations Between Media Use Factors and Mental Health

The results of multiple linear regression showed significant relationships between the seven media factors as regressors and anxiety (GAD-7 total score) as the explained variable (Table 3). The media factors news aversion, social media practice, news reading, internalized use factors, and COVID-19 news exposure predicted higher levels of anxiety, with social media practice having the strongest effect size. The associations were significant in both observation periods, except for news reading, which was found as a significant predictor only in the second wave of the survey. Therefore, most of the factors were found to be stable over time, while the strength of the associations increased in most cases. In particular, associations between three media factors (COVID-19-news use, social media practice, and infotainment) and anxiety were considerably stronger in the second wave than in the first wave. We also found two media factors, infotainment and reading habits, that predicted lower anxiety levels in both waves, although reading habits had much smaller effect sizes compared to those of the other factors. The strength of the association between the factor infotainment and anxiety considerably increased in the second wave.

The multiple linear regression model for depression (total PHQ-8 score as explained variable) identified identical predictors as the model of anxiety (Table 4), except for news reading, which was not identified as a significant predictor in depression. The media factors also proved to be fairly stable over time. In the first wave of the survey, the media factors news aversion, social media practice, and internalized use, and COVID-19 news exposure predicted higher levels of depression, and the same was true in the second wave. Again, the same media factors as in the models explaining anxiety predicted lower rates of depression: infotainment in both waves and reading habits only in the second wave. The effect size of reading habits was smaller than that of the other media predictors. Social media practice and infotainment had the strongest effect sizes among all media factors in the second wave of the survey, following a considerable increase compared to the first wave of the survey.

**Table 3:** Results of the multiple linear regression model for anxiety.

Variable	First wave of the survey (*F*=25.77; *df*=1764; *R*^*2*^=0.1491; *P*<.001)					Second wave of the survey (*F*=37.67; *df*=1764; *R*^*2*^=0.204; *P*<.001)		
		Coefficient (SE)	*t* value	*P* value	Coefficient (SE)		*t* value	*P* value
Intercept		1.484 (0.176)	8.449	<.001	1.288 (0.146)		8.829	<.001
Conscientious public news use (MF^a^1)		–0.016 (0.037)	–0.422	.67	0.041 (0.077)		0.532	.59
News aversion (MF2)		0.186 (0.032)	5.803	<.001	0.147 (0.070)		2.106	.04
News reading (MF3)		0.053 (0.033)	1.589	.11	0.092 (0.041)		2.257	.02
Social media practice (MF4)		0.263 (0.034)	7.773	<.001	0.584 (0.077)		7.618	<.001
Infotainment (MF5)		–0.071 (0.035)	–2.026	.04	–0.643 (0.166)		–3.880	<.001
Internalized use (MF6)		0.263 (0.033)	8.038	<.001	0.282 (0.067)		4.224	<.001
Practical use (MF7)		0.015 (0.032)	0.457	.65	0.193 (0.114)		1.689	.09
Media news exposure		–0.0004 (0.0004)	–0.914	.36	–0.001 (0.0004)		–1.263	.21
COVID-19 news exposure		0.090 (0.040)	2.249	.03	0.280 (0.035)		7.951	<.001
Reading habits		–0.042 (0.021)	–1.990	.05	–0.061 (0.020)		–2.969	.003
Age		–0.011 (0.002)	–6.011	<.001	–0.016 (0.002)		–8.440	<.001
Gender (women)		0.123 (0.061)	2.031	.04	0.203 (0.057)		3.584	<.001

^a^MF: media factor.

**Table 4:** Results of the multiple linear regression model for depression.

Variable	First wave of the survey (*F*=25.34; *df*=1764; *R*^2^=0.147; *P*<.001)					Second wave of the survey (*F*=30.78; *df*=1764; *R*^2^=0.173; *P*<.001)		
		Coefficient (SE)	*t* value	*P* value	Coefficient (SE)		*t* value	*P* value
Intercept		1.273 (0.178)	7.137	<.001	1.533 (0.150)		10.223	<.001
Conscientious public news use (MF^a^1)		0.001 (0.038)	0.025	.98	–0.100 (–0.099)		–1.259	.21
News aversion (MF2)		0.159 (0.032)	4.905	<.001	0.187 (0.072)		2.609	.009
News reading (MF3)		0.048 (0.034)	1.431	.15	0.048 (0.042)		1.161	.25
Social media practice (MF4)		0.265 (0.034)	7.723	<.001	0.503 (0.079)		6.368	<.001
Infotainment (MF5)		–0.122 (0.036)	–3.435	.001	–0.471 (0.170)		–2.767	.006
Internalized use (MF6)		0.229 (0.033)	6.901	<.001	0.254 (0.069)		3.696	<.001
Use of practical news (MF7)		–0.005 (0.033)	–0.165	.87	0.019 (0.117)		0.164	.87
Media news exposure		–0.00037 (0.0004099)	–0.919	.36	–0.0002904 (0.0004214)		–0.689	.49
COVID-19 news exposure		0.161 (0.041)	3.963	<.001	0.233 (0.036)		6.445	<.001
Reading habits		–0.021 (0.021)	–0.993	.32	–0.042 (0.021)		–1.997	.05
Age		–0.009 (0.002)	–4.978	<.001	–0.015 (0.002)		–7.856	<.001
Gender (women)		0.199 (0.061)	3.239	.001	0.259 (0.058)		4.439	<.001

^a^MF: media factor.

## Discussion

### Principal Findings

The findings of our longitudinal representative study showed that the symptoms of anxiety and depression of the Czech population increased in the late phase of the COVID-19 lockdown compared to the early phase. We further identified the negative role of media coverage of the COVID-19 pandemic in self-reported measures of mental health in both the early and late phases of the pandemic lockdown in Czechia. Moreover, other specific media practices were revealed as either risk or protective factors for mental health. Most of these media factors were found to be fairly stable and long-lasting characteristics associated with mental health, and some of them were more pronounced in the second observation period.

### Impact of the Long Lockdown on Mental Health

Our finding of an overall increase of anxiety and depression symptoms between the two observation periods shows that the pandemic lockdown spanning 5 months had a negative impact on mental health of the Czech population, even when the objective measures of the epidemiological situation (daily incidence and mortality) in the two periods were comparable or slightly more favorable in the late phase compared to the early phase of the lockdown. Therefore, our data strongly suggest that severe restrictions and continuous exposure to the collective trauma did not result in a strengthening of resilience but rather in ongoing traumatization. This is in line with several previous longitudinal studies, which showed a higher level of mental health problems in response to a long, drawn-out lockdown, comparing an advanced stage of lockdown to its initial stage [27-29]. Our results also confirm a previous finding that the strict lockdown measures played a more significant role in mental health than the epidemiological situation itself [30]. The long period of the health emergency and restrictions on the normal life of society without any clear prospect of an end may have fostered frustration, uncertainty, fear of infection or death, loss of employment, and reduced household incomes, which have previously been associated with an increase in long-term psychological problems (anxiety, depression, insomnia, or posttraumatic stress symptoms) [31,32]. Therefore, we conclude that human well-being is at long-term risk during a long pandemic crisis addressed by a lockdown.

Our next finding, which associated higher levels of anxiety and depression with the female gender in both observation periods, is in accordance with previous studies on mental health during the COVID-19 pandemic [33-35]. One possible explanation for this may be related to the closing of schools during the protracted lockdown, when women largely had to bear with the additional burden of home childcare and teaching. The association of higher rates of anxiety and depression with younger ages that we found in both observation periods is also in line with other studies [36]. Possibly, younger people may be more affected by the pandemic restrictions, resulting in multifaced uncertainties affecting their lives as well as social isolation from their peers.

### Media Factors Associated With Poorer Mental Health

Our results confirmed a growing body of literature suggesting the negative impact of exposure to COVID-19 reports on mental health [3,9,11]. Additionally, by showing that exposure to specific COVID-19–related content (*COVID-19 news exposure*) predicted increased levels of anxiety and depression unlike exposure to news reports in general (*media news exposure*), we confirmed that not all media news consumption is solely negative for mental health, but specifically the consumption of topics related to a current (collective) stressor. Interestingly, the comparison of the predictive powers of COVID-19 news exposure between the two phases of the lockdown did not confirm a habituation to COVID-19 media reports. By contrast, exposure to COVID-19 news remained a stable predictor of depression, and the association strengthened for anxiety in the late phase of the lockdown. We may speculate that the missing association between COVID-19 news exposure and anxiety levels in the early phase of the pandemic crisis may be due to the widespread conviction that the forthcoming wave of the epidemic would again be contained without major consequences and with a minimum of casualties, similar to the first peak in the pandemic in Czechia in the spring of 2020. However, after 5 months of continuous daily presentation of the life-threatening situation and unclear solutions, the exposure to COVID-19 news reports became a stronger risk factor for mental health. In summary, our finding provides further support for understanding COVID-19 media coverage as a traumatic stressor acting in the long term, as suggested by research associating the frequent use of media reports on COVID-19 with secondary traumatization [37,38].

In our search for specific media practices and their potential negative or protective role in mental health, we identified several predictors of mental health, which we first extracted from our comprehensive MUQ via a data-driven approach. The strongest predictor of mental health among all other media or demographic predictors was the media factor that we call *social media practice in news consumption*. The interconnection of three specific aspects of media use in this factor—use of social media as a news source, reading comments under web news articles, and perceived stress from the media—points to an existence of a media use practice linked to the social media environment, which prompts its users to read comments on posts. This social media practice seems to apply to news consumption as well, prompting media users to read comments under web news articles. Importantly, the social media practice factor suggests that such a media practice, including reading anonymized and therefore often highly negatively balanced, comments under web news articles [39], is connected to a subjective perception of stress induced by the media, which is in line with our other finding associating this factor to increased levels of anxiety and depression. These results are in accordance with previous research connecting social media use to stress and other mental health problems [14,40]. Identifying social media practice for news as a risk factor for mental health possibly links to the pandemic-related misinformation overload on Czech social media [41], and the spread of fear, panic, frustration, and other negative emotions [41-44], since social media has been confirmed to be a perfect platform for “emotional contagion” [45]. The increase in predictive powers of the social media factor for both anxiety and depression in the late phase of the lockdown (spring 2021) may be linked to the vaccination of the population, which began in Czechia at the end of December 2020. The vaccination rollout triggered an avalanche of misinformation and conspiracies on social media, resulting in a division of society into vaccine supporters and refusers. Moreover, the increased strength of the relationship may have been due to the prolonged exposure to traumatic content and emotive comments that circulated on social media during the long-lasting crisis.

The media use factor that we call *internalized use*, describing the personal engagement with information learned from media news, has not yet been described in the literature. This data-driven factor, grouping several indicators of a deep immersion in the media news [46], including ruminations on news content or its impact on one’s own decision-making and action, predicted increased anxiety and depression in both waves, and may therefore be considered another risk factor for mental health. Hence, we confirmed that not only exposure to traumatic news is of significance but also the extent to which users internally engage with it and the importance they give to it. This finding offers possible ways to mitigate the negative impact of traumatic news on mental health. Psychotherapeutic techniques developed to stop negative thinking may be used to practice deliberate avoidance of traumatic topics in one’s thoughts and further emotional engagement in the traumatic news.

Our next result that the *news aversion* factor predicted higher rates of anxiety and depression in both phases of the lockdown corresponds with the findings of a few studies that link mistrust in the media to mental health problems [12,47]. However, in addition to mistrust, the news aversion factor was constructed of further negative attitudes to media news (frustration, annoyance, avoidance). Negative attitudes to the information source associated with poorer mental health may be interpreted as a reaction to a failure to cope with ambiguous or frequently changing information, along with uncertainty of the epidemic situation and its possible consequences [46]. Avoidant reactions to media news contained in the news aversion factor may be seen as analogous to the depressive symptom of withdrawal from the external world, which is considered an avoidance response to a problematic stimulus. This factor must not be overlooked, as other types of problematic behavior have been related to negative attitudes to the media. Together with mistrust in public institutions, avoidant behavior has been previously connected to an inclination to extreme views or even extremism and aggression [48]. Previous research has also suggested that aversion is associated to detrimental media behavior such as searching for antisystem websites, endorsement of conspiracy theories [49], or following and sharing disinformation and extreme views on social media [50].

The *news reading* factor was revealed in our data to be a media practice common to users of a wide range of online and print media, whereas the use of audio/audiovisual media outlets contributed to other factors (conscientious public news use, infotainment). An association between the practice of reading and poorer mental health was found; however, this was only the case for anxiety in the late phase of the lockdown. This may be due to the escalating polarization of views on vaccination, which was more apparent in online media news than other media types, because of the greater diversity of views represented in the online environment, including the antisystem media. At the time of the second wave of our survey, the process of vaccination against COVID-19 was in full swing in Czechia, and so was the fear-inducing news published by the antisystem media [41].

In summary, we may interpret our media factors that played a negative role in mental health as indicators of four levels of the spread of negative mental states from traumatic news content: (1) behavioral level of the consumption of negative information contained in COVID-19 media news reports (*COVID-19 news exposure*); (2) subjective level of immersion in media news and its internalization; (3) level of attitudes, including refusal of the media, possibly due to negative information overload, manifesting in aversive reactions; and (4) a specific platform of a social media environment that strengthens present emotional biases, and multiplies and accelerates the process of spreading of negative mental states in society [51,52].

### Media Factors Associated With Improvement of Mental Health

The association between the *infotainment* factor, grouping consumption of entertainment topics and consumption of news from commercial media, and improvement of mental health contradicts some of the previous research [3], which found negative effects of using commercial media on mental health during the COVID-19 pandemic, although our infotainment factor contained further indicators of media use (interest in entertainment topics). Sports and show business or even crime news with an air of police drama may have distracted participants from otherwise stressful news focused almost exclusively on the COVID-19 pandemic. The discrepancy with the previous study [3] may be further due to the fact that our survey took place in a different situation (the previous study’s data were collected during the first 2 weeks of April 2020), and also possibly due to the different characteristics of Czech commercial media.

The second protective factor found in our data, *reading habits* (contextual in-depth reading vs only headline browsing), did not have as a pronounced effect on mental health as other media factors (especially in depression); however, we still consider it an important finding, suggesting a possible remedy for the consumption of traumatic news. Receiving and processing contextual information may possibly be associated with “cold” cognition (ie, information processing in the absence of emotional influence), which is crucial to analytical thinking, whereas headline browsing may be associated with “hot” cognition (ie, fast and emotional). Impairment of “cold” cognition has previously been connected to both depression and anxiety [53,54]. Even though the method used in this study does not enable inferring causality, we suggest that contextual reading, as a slower and more analytical approach to consuming media news, thereby nurturing “cold” cognition, may lead to a more adaptive processing of a stressor, and may therefore be considered a protective factor to mental health. The weaker association in the early phase of the lockdown may be interpreted to indicate that quick headline browsing and employing “hot” cognition may be more damaging in the long run in terms of coping with the persisting collective stressor spread by the media.

### Conclusions

The 5 months of the second pandemic lockdown in Czechia had a negative impact on mental health, and caused an increase in some of the negative, as well as positive, media use effects. Our results suggest that exposure to COVID-19 news played a negative role in mental health during the early and particularly the late phase of the lockdown in Czechia, while exposure to news media as such did not. The social media practice in news consumption was the strongest predictor of exacerbated mental health symptoms of all the examined factors, particularly in the late phase of the lockdown. Additionally, news media use characterized by internalization of information learned from the news, as well as negative attitudes to media news, were associated with higher levels of anxiety and depression. Stability or strengthening of associations between these media factors and exacerbation of mental health symptoms in the late phase of the lockdown leads us to the conclusion that these factors had a long-term or even increasing negative effect on mental health. By contrast, the use of infotainment as well as the in-depth and contextual practice of reading news articles were related to lower rates of anxiety and depression, thereby perhaps easing the burden of the crisis in terms of media use. These protective media use practices considerably strengthened in the late period of the lockdown.

## Appendix

Multimedia Appendix 1 Percentage of answers in media use questionnaire.

Multimedia Appendix 2 Factor loadings of media factors resulting from exploratory factor analysis.

## Abbreviations

EFA: exploratory factor analysis
GAD-7: Generalized Anxiety Disorder Questionnaire
MUQ: Media Use Questionnaire
PHQ-8: Patient Health Questionnaire Depression Scale
