# Supplementary material for: Impact of a Long Lockdown on Mental Health and the Role of Media Use: Web-Based Survey Study
Source: JMIR Ment Health. 2022 Jun 28;9(6):e36050. doi: 10.2196/36050 (PMC9277533; doi:10.2196/36050)
Supplement: Multimedia Appendix 1 [file mental_v9i6e36050_app1.docx]

Multimedia Appendix 1. Percentage of answers in media use questionnaire

| Items | Wave | Never | Less than once a week | | | Once a week | | | | | Several times in week | | | | One or two times a day | | | Several times a day | | | | | | | Overall absolute change between waves | | | | |  |  |
| --- | --- | --- | --- | --- | --- | --- | --- | --- | --- | --- | --- | --- | --- | --- | --- | --- | --- | --- | --- | --- | --- | --- | --- | --- | --- | --- | --- | --- | --- | --- | --- |
| **Media categories** |  |  |  | | |  | | | | |  | | | |  | | |  | | | | | | |  | | | | |  |  |
| Public | 1 | 8,55 | 17,33 | | | 11,37 | | | | | 21,38 | | | | 27,46 | | | 13,90 | | | | | | | 12.37 | | | | |  |  |
|  | 2 | 12,49 | 16,09 | | | 8,10 | | | | | 19,70 | | | | 29,36 | | | 14,24 | | | | | | |  |  |  |  |  |  |  |
| Mainstream | 1 | 13,96 | 33,26 | | | 35,62 | | | | | 13,90 | | | | 3,04 | | | 0,23 | | | | | | | 12.14 | | | | |  |  |
|  | 2 | 19,75 | 33,48 | | | 31,68 | | | | | 12,16 | | | | 2,64 | | | 0,28 | | | | | | |  | | | | |  |  |
| Commercial | 1 | 11,82 | 31,06 | | | 36,69 | | | | | 17,22 | | | | 3,04 | | | 0,17 | | | | | | | 18.46 | | | | |  |  |
|  | 2 | 16,77 | 35,23 | | | 30,33 | | | | | 14,52 | | | | 2,87 | | | 0,28 | | | | | | |  | | | | |  |  |
| Anti-system | 1 | 63,25 | 20,71 | | | 6,42 | | | | | 5,97 | | | | 3,1 | | | 0,56 | | | | | | | 3.59 | | | | |  |  |
|  | 2 | 63,03 | 19,70 | | | 7,09 | | | | | 5,40 | | | | 3,71 | | | 1,07 | | | | | | |  | | | | |  |  |
| Official public sources | 1 | 39,67 | 36,63 | | | 10,80 | | | | | 8,55 | | | | 3,49 | | | 0,84 | | | | | | | 10.12 | | | | |  |  |
|  | 2 | 44,63 | 32,75 | | | 10,75 | | | | | 7,65 | | | | 3,60 | | | 0,62 | | | | | | |  | | | | |  |  |
| Opinion online newspapers | 1 | 46,54 | 26,0 | | | 8,27 | | | | | 11,08 | | | | 5,51 | | | 2,59 | | | | | | | 7.42 | | | | |  |  |
|  | 2 | 48,45 | 23,07 | | | 9,0 | | | | | 10,30 | | | | 6,36 | | | 2,81 | | | | | | |  | | | | |  |  |
| Foreign news | 1 | 59,48 | 25,49 | | | 7,54 | | | | | 5,23 | | | | 1,80 | | | 0,45 | | | | | | | 5.62 | | | | |  |  |
|  | 2 | 61,17 | 23,47 | | | 6,75 | | | | | 5,23 | | | | 2,53 | | | 0,84 | | | | | | |  | | | | |  |  |
| **Media types** |  |  |  | | |  | | | | |  | | | |  | | |  | | | | | | |  | | | | |  |  |
| Audio/audiovisual | 1 | 4,39 | 14,63 | | | 31,79 | | | | | 33,71 | | | | 14,24 | | | 1,24 | | | | | | | 12.38 | | | | |  |  |
|  | 2 | 6,92 | 18,01 | | | 30,95 | | | | | 29,54 | | | | 13,06 | | | 1,52 | | | | | | |  | | | | |  |  |
| Print/internet | 1 | 17,67 | 52,79 | | | 24,48 | | | | | 4,45 | | | | 0,56 | | | 0,06 | | | | | | | 66.63 | | | | |  |  |
|  | 2 | 3,88 | 33,26 | | | 41,42 | | | | | 16,94 | | | | 4,22 | | | 0,28 | | | | | | |  | | | | |  |  |
| **News content** |  | Not interested at all | | | Rather not interested | | | | | Rather interested | | | | | Very interested | | | | | | |  | | |  | | | | |  |  |
| Politics | 1 | 8,38 | | | 22,28 | | | | | 48,84 | | | | | 20,48 | | | | | | |  | | 14.52 | | | | |  |  |  |
|  | 2 | 12,44 | | | 25,49 | | | | | 45,24 | | | | | 16,83 | | | | | | |  | |  | | | | |  |  |  |
| Economics | 1 | 12,83 | | | 29,60 | | | | | 45,81 | | | | | 11,76 | | | | | | |  | | 14.98 | | | | |  |  |  |
|  | 2 | 16,49 | | | 33,43 | | | | | 40,63 | | | | | 9,45 | | | | | | |  | |  | | | | |  |  |  |
| Covid-19 | 1 | 3,83 | | | 14,55 | | | | | 47,55 | | | | | 34,50 | | | | | | |  | | 19.94 | | | | |  |  |  |
|  | 2 | 7,48 | | | 20,20 | | | | | 48,0 | | | | | 24,31 | | | | | | |  | |  | | | | |  |  |  |
| Entertainment | 1 | 33,82 | | | 39,79 | | | | | 23,66 | | | | | 2,76 | | | | | | |  | | 8.14 | | | | |  |  |  |
|  | 2 | 37,87 | | | 38,04 | | | | | 22,17 | | | | | 1,91 | | | | | | |  | |  | | | | |  |  |  |
| Culture | 1 | 19,36 | | | 44,40 | | | | | 32,98 | | | | | 3,26 | | | | | | |  | | 10.24 | | | | |  |  |  |
|  | 2 | 24,48 | | | 44,29 | | | | | 28,59 | | | | | 2,64 | | | | | | |  | |  | | | | |  |  |  |
| Sport | 1 | 36,86 | | | 27,86 | | | | | 22,90 | | | | | 12,38 | | | | | | |  | | 4.51 | | | | |  |  |  |
|  | 2 | 35,17 | | | 27,29 | | | | | 23,69 | | | | | 13,84 | | | | | | |  | |  | | | | |  |  |  |
| Science | 1 | 19,41 | | | 34,27 | | | | | 38,60 | | | | | 7,71 | | | | | | |  | | 7.66 | | | | |  |  |  |
|  | 2 | 23,24 | | | 33,48 | | | | | 36,18 | | | | | 7,09 | | | | | | |  | |  | | | | |  |  |  |
| Crime | 1 | 23,86 | | | 38,72 | | | | | 33,71 | | | | | 3,71 | | | | | | |  | | 9.9 | | | | |  |  |  |
|  | 2 | 28,59 | | | 38,94 | | | | | 29,21 | | | | | 3,26 | | | | | | |  | |  | | | | |  |  |  |
| Transport | 1 | 22,17 | | | 36,47 | | | | | 35,23 | | | | | 6,13 | | | | | | |  | | 14.52 | | | | |  |  |  |
|  | 2 | 29,43 | | | 36,35 | | | | | 29,94 | | | | | 4,28 | | | | | | |  | |  | | | | |  |  |  |
| Weather | 1 | 3,88 | | | 13,96 | | | | | 49,80 | | | | | 32,36 | | | | | | |  | | 11.7 | | | | |  |  |  |
|  | 2 | 7,03 | | | 16,32 | | | | | 50,14 | | | | | 26,51 | | | | | | |  | |  | | | | |  |  |  |
| Environment | 1 | 15,53 | | | 37,65 | | | | | 39,45 | | | | | 7,37 | | | | | | |  | | 10.92 | | | | |  |  |  |
|  | 2 | 20,26 | | | 38,38 | | | | | 36,07 | | | | | 5,29 | | | | | | |  | |  | | | | |  |  |  |
| Health | 1 | 9,12 | | | 27,97 | | | | | 49,24 | | | | | 13,67 | | | | | | |  | | 16.88 | | | | |  |  |  |
|  | 2 | 14,35 | | | 31,18 | | | | | 44,57 | | | | | 9,90 | | | | | | |  | |  | | | | |  |  |  |
|  |  | Never | Very rarely | | | | Sometimes | | | | | Quite often | | | | | Very often | | | | |  | | |  | | | | |  |  |
| **Reading comments** | 1 | 9,57 | 27,01 | | | | 40,97 | | | | | 16,26 | | | | | 6,19 | | | | |  | | | 10.35 | | | | |  |  |
|  | 2 | 10,24 | 31,51 | | | | 37,87 | | | | | 14,91 | | | | | 5,46 | | | | |  | | |  | | | | |  |  |
| **Social media as an information source** | 1 | 15,64 | 21,22 | | | | 26,39 | | | | | 13,56 | | | | | 11,54 | | | | |  | | | 20.66 | | | | |  |  |
|  | 2 | 18,57 | 22,28 | | | | 29,43 | | | | | 22,68 | | | | | 7,03 | | | | |  | | |  | | | | |  |  |
| **Perceived impact of news media** |  | Completely disagree | | | | Rather disagree | | | | | Rather agree | | | | | Completely agree | | | | |  | |  | | |  | | | |  |  |
| Stress | 1 | 5,40 | | | | 41,92 | | | | | 45,98 | | | | | 6,70 | | | | |  | |  | | | 16.2 | | | | | |
|  | 2 | 11,03 | | | | 39,39 | | | | | 40,41 | | | | | 9,17 | | | | |  | |  | | |  | | | | | |
| Internalization of news | 1 | 3,04 | | | | 30,84 | | | | | 60,78 | | | | | 5,35 | | | | |  | |  | | | 28.25 | | | | | |
| **Positive appreciation of news** | 2  1  2 | 8,05  5,35  11,25 | | | | 39,95  20,99  26,00 | | | | | 48,96  63,14  54,92 | | | | | 3,04  10,52  7,82 | | | | |  | |  | | | 21.83 | | | | | |
| **Reading habits** |  | Don’ t read news | | Read only headlines | | | | Read only title and first lines | | | | | | Read only about half of article | | | | | | Read full article | | | | | | | |  | | |  |
|  | 1 | 7,09 | | 6,53 | | | | 18,46 | | | | | | 14,69 | | | | | | 46,31 | | | | | | | | 15.01 | | |  |
|  | 2 | 9,62 | | 8,27 | | | | 21,22 | | | | | | 15,70 | | | | | | 39,34 | | | | | | | |  | | |  |
| **Attitudes to media** |  | Strongly disagree | | Disagree | | | | | Rather disagree | | | | Rather agree | | | Agree | | | Strongly agree | | | | | | | |  | | |  |  |
| Frustration | 1 | 2,87 | | 11,20 | | | | | 27,29 | | | | 37,03 | | | 12,94 | | | 8,67 | | | | | | | | 7.31 | | |  |  |
|  | 2 | 3,32 | | 10,52 | | | | | 24,76 | | | | 37,76 | | | 12,49 | | | 11,14 | | | | | | | |  | | |  |  |
| Lack of concentration | 1 | 10,24 | | 34,38 | | | | | 35,00 | | | | 14,35 | | | 4,84 | | | 1,18 | | | | | | | | 7.99 | | |  |  |
|  | 2 | 10,35 | | 30,39 | | | | | 35,68 | | | | 16,43 | | | 5,29 | | | 1,86 | | | | | | | |  | | |  |  |
| Lack of time | 1 | 14,01 | | 36,52 | | | | | 27,80 | | | | 14,18 | | | 4,56 | | | 2,93 | | | | | | | | 7.31 | | |  |  |
|  | 2 | 13,45 | | 33,65 | | | | | 30,50 | | | | 15,08 | | | 4,61 | | | 2,70 | | | | | | | |  | | |  |  |
| Annoyance | 1 | 1,80 | | 8,72 | | | | | 17,56 | | | | 38,66 | | | 16,94 | | | 16,32 | | | | | | | | 4.05 | | |  |  |
|  | 2 | 2,48 | | 8,33 | | | | | 15,93 | | | | 38,83 | | | 17,67 | | | 16,77 | | | | | | | |  | | |  |  |
| Stress avoidance | 1 | 5,76 | | 16,83 | | | | | 20,09 | | | | 28,53 | | | 16,04 | | | 12,72 | | | | | | | | 8.26 | | |  |  |
|  | 2 | 7,15 | | 13,11 | | | | | 21,10 | | | | 30,16 | | | 15,64 | | | 12,83 | | | | | | | |  | | |  |  |
| Lack of interest | 1 | 15,42 | | 33,15 | | | | | 29,71 | | | | 12,21 | | | 5,80 | | | 3,71 | | | | | | | | 10.48 | | |  |  |
|  | 2 | 13,73 | | 29,94 | | | | | 30,78 | | | | 15,87 | | | 5,46 | | | 4,22 | | | | | | | |  | | |  |  |
| Mistrust | 1 | 4,05 | | 13,96 | | | | | 29,54 | | | | 26,28 | | | 12,94 | | | 13,22 | | | | | | | | 6.18 | | |  |  |
|  | 2 | 5,80 | | 15,25 | | | | | 29,60 | | | | 25,72 | | | 10,75 | | | 12,89 | | | | | | | |  | | |  |  |
