# Supplementary material for: Impact of a Long Lockdown on Mental Health and the Role of Media Use: Web-Based Survey Study
Source: JMIR Ment Health. 2022 Jun 28;9(6):e36050. doi: 10.2196/36050 (PMC9277533; doi:10.2196/36050)
Supplement: Multimedia Appendix 2 [file mental_v9i6e36050_app2.docx]

Multimedia Appendix 2. Factor loadings of media factors resulting from exploratory factor analysis

| Items | MF1 | MF2 | MF3 | MF4 | MF5 | MF6 | MF7 |
| --- | --- | --- | --- | --- | --- | --- | --- |
| Lack of interest | **-.54** | .52 | -.09 | -.12 | -.07 | -.22 | -.13 |
| Lack of time | **-.53** | .43 | -.01 | -.13 | -.09 | -.08 | -.05 |
| Politics | **.52** | -.20 | .31 | .12 | -.01 | .28 | .19 |
| Economics | **.41** | -.09 | .39 | .01 | -.03 | .22 | .30 |
| Public | **.32** | -.19 | .19 | -.11 | .17 | .26 | .12 |
| Mistrust | -.03 | **.65** | -.03 | .04 | -.10 | -.34 | -.03 |
| Frustration | .03 | **.65** | -.05 | .19 | .02 | -.01 | -.03 |
| Stress avoidance | -.22 | **.64** | -.09 | .03 | .00 | -.07 | -.05 |
| Annoyance | -.08 | **.63** | -.10 | .01 | -.11 | -.12 | -.09 |
| Lack of concentration | -.33 | **.42** | -.01 | .04 | .07 | .02 | -.01 |
| Opinion online newspapers | .04 | -.08 | **.68** | .07 | .00 | .13 | -.04 |
| Foreign news | .01 | -.04 | **.63** | .00 | .00 | .01 | .05 |
| Mainstream | .13 | -.17 | **.60** | .06 | .11 | .22 | .00 |
| Anti-system | .11 | .11 | **.50** | .14 | .07 | -.11 | .00 |
| Official public sources | .00 | -.08 | **.41** | .11 | .09 | .01 | .15 |
| Perceived stress | .06 | .31 | .05 | **.75** | .08 | .20 | .09 |
| Reading comments | .15 | .02 | .23 | **.67** | .00 | -.01 | .06 |
| Social media as an information source | -.07 | .01 | .08 | **.39** | .18 | .04 | .05 |
| Commercial | .04 | -.02 | .11 | .14 | **.59** | .03 | .04 |
| Entertainment | -.16 | -.09 | -.04 | .20 | **.48** | .12 | .26 |
| Crime | .10 | -.06 | .04 | .14 | **.46** | -.02 | .44 |
| Sport | .23 | .01 | .14 | -.17 | **.32** | .04 | .22 |
| Internalization of news | .21 | -.16 | .11 | .18 | .10 | **.77** | .16 |
| Positive appreciation of news | .09 | -.20 | .04 | .06 | .01 | **.66** | .14 |
| Environment | -.02 | -.10 | .10 | .09 | .00 | .08 | **.74** |
| Health | .03 | -.10 | .05 | .23 | .07 | .17 | **.65** |
| Transport | .13 | .01 | .06 | -.06 | .40 | -.02 | **.47** |
| Weather | .12 | .05 | -.09 | -.01 | .13 | .05 | **.45** |
| Culture | -.01 | -.12 | .16 | .03 | .25 | .15 | **.44** |
| Science | .24 | -.06 | .35 | -.16 | .00 | -.03 | **.41** |
